# Ultra-sensitive fluorescence-activated droplet single-cell sorting based on Tetramer-HCR-EvaGreen amplification

**DOI:** 10.1038/s41378-024-00861-8

**Published:** 2025-01-16

**Authors:** Long Chen, Yi Xu, Lele Zhou, Ding Ma, Rong Zhang, Yifan Liu, Xianqiang Mi

**Affiliations:** 1 Shanghai Institute of Microsystem and Information Technology, Chinese Academy of Sciences, Shanghai, 200050 China; 2 Shanghai Advanced Research Institute, Chinese Academy of Sciences, Shanghai, 201210 China; 3 University of Chinese Academy of Sciences, Beijing, 100049 China; 4 School of Physical Science and Technology, ShanghaiTech University, Shanghai, 201210 China; 5 Shanghai Clinical Research and Trial Center, Shanghai, 201210 China; 6 State Key Laboratory of Advanced Medical Materials and Devices, ShanghaiTech University, Shanghai, 201210 China; 7 School of Physics and Optoelectronic Engineering Hangzhou Institute for Advanced Study, University of Chinese Academy of Sciences, Chinese Academy of Sciences, Hangzhou, 310024 China

**Keywords:** Microfluidics, Nanoscience and technology

## Abstract

The current single-cell analysis technologies such as fluorescence-activated cell sorting (FACS) and fluorescence-activated droplet sorting (FADS) could decipher the cellular heterogeneity but were constrained by low sorting performance and cell viability. Here, an ultra-sensitive single-cell sorting platform has been developed by integrating the FADS technology with Tetramer-HCR-EvaGreen (THE) fluorescence signal amplification. The THE system produced much higher fluorescence signal than that of the single Tetramer or Tetramer-HCR signal amplification. Upon application to target MCF-7 cells, the platform exhibited high efficacy and selectivity while maintaining more than 95% cell viability. The THE-FADS achieved sorting efficiencies of 55.5% and 50.3% with purities of 91% and 85% for MCF-7 cells in PBS solutions and simulated serum samples, respectively. The sorted MCF-7 cells showed similar proliferation together with CK19 and EGFR mRNA expression compared with the control cells. The established THE-FADS showed the promising prospects to cellular heterogeneity understanding and personalized medicine.

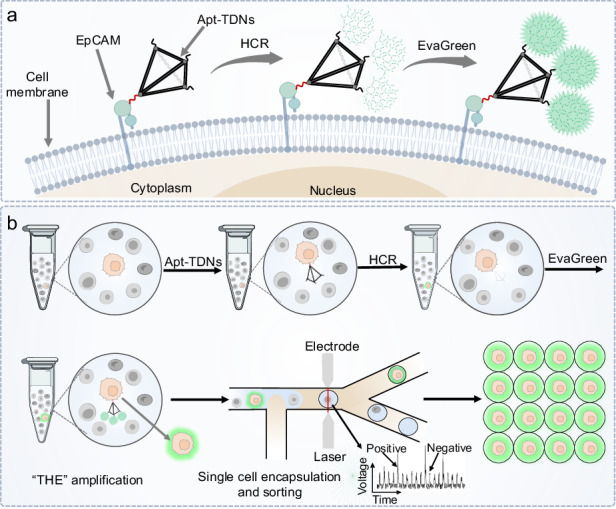

## Introduction

In recent years, single-cell analysis has gained increasing interest due to its ability to investigate cellular heterogeneity, which could substantially enhance our understanding of carcinogenesis and tumor evolution^1,2^. Initial advancements in single-cell analysis were predominately driven by fluorescence-activated cell sorting (FACS), which offers benefits such as high throughput, standardization, and multi-parameter analysis capabilities^3^. However, the limitations such as dependency on costly antibodies and detrimental impact on cell viability constrain its practical applications^3,4^.

Over the past few years, droplet microfluidics has been rapidly developed, playing a significant role in high-throughput single-cell analysis and related biomedical research, with the advantages of high throughput, minimum sample and reagent consumption, and less reaction time^5–7^. Droplet manipulation techniques such as merging, splitting, sorting, and liquid injection have gained significant advances and improvements^8^. Fluorescence-activated droplet sorting (FADS) has been widely employed to isolate bio-targets encapsulated in droplets from a continuous stream^9–11^. Different from FACS, FADS employs confined and separated nanoliter or picolitre oil-emulsified droplets with biological samples being encapsulated into compartmentalized space, which is quite appropriate for the subsequent single-cell analysis^12^. Recently, FADS has rapidly emerged as a powerful tool for ultra-high-throughput single-cell screening. For instance, Gerard et al.^13^utilized antibody-coated magnetic beads to capture immunoglobulin G from single B cells in microdroplets, enabling fluorescence-based droplet sorting and functional antibody screening. To date, most FADS-based technologies targeted intracellular biomolecules (protein, nucleic acid, etc.)^14–16^or cell-secreting biomolecules (protein, nucleic acid, etc.)^11,13,17^. These FADS strategies suffer from complex operations such as in-droplet cell lysis, cell transfection and stimulation, which would seriously affect cell viability. Furthermore, the sensitivity of conventional FADS was primarily constrained by the low-intensity signals of fluorescent antibody or aptamers labelling^13^. These drawbacks hinder the broader potential application.

In recent years, rationally assembled DNA nanostructures, such as DNA tetrahedra, have been applied in bioimaging^18–20^, biosensing^21–23^, bio-computation^24^, and drug delivery^25^. The DNA tetrahedra possesses unique features such as structural rigidity, nanoscale controllability, and excellent biocompatibility^26–28^. Furthermore, it is relatively simple to attach nucleic acid probes to the tetrahedra’s edges for specific targeting. To achieve ultra-sensitive detection with DNA nanostructure-based approaches, various signal amplification strategies, including polymerase chain reaction^29^, hybridization chain reaction (HCR)^30–32^, rolling circle amplification (RCA)^33–35^, and DNA strand displacement amplification (SDA)^36–38^ have been utilized. Combining the DNA tetrahedra with HCR amplification, a feasible and high-efficiency signal amplification could be produced. For example, Wang et al. ^39^ developed an aptamer-DNA tetrahedra-specific recognition and HCR signal amplification technology for the capturing of CTCs with high efficiency. Nucleic acid intercalating dyes such as EvaGreen^40,41^ or SYBR Green I^42,43^, known for their advantages of cost-effectiveness, low toxicity, high stability and high selectivity, have also been widely employed to augment the fluorescence signal. For example, Zhao et al. ^43^ developed a high-sensitivity Hela cell fluoresce signal amplifier by functionalizing a DNA dendrimer with the sgc8 aptamer and SYBR Green I dye.

**Scheme 1:**
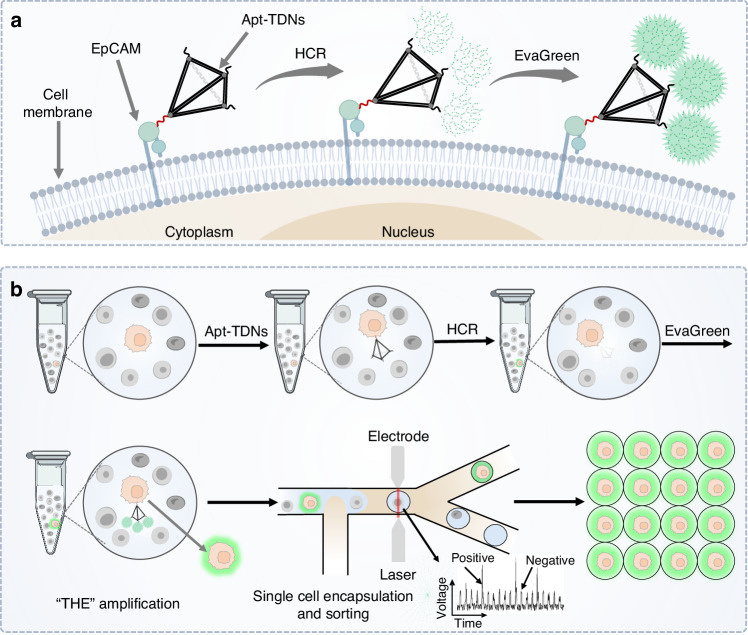
Schematic representation of ultra-sensitive single-cell sorting through the THE-FADS. **a** The MCF-7 cells were illuminated using the THE signal amplification system. The EpCAM protein was located on the membrane of MCF-7 cells. The Apt-TDNs could bind EpCAM, and then the HCR was initiated to form three dendritic DNA structures with fluorescence signal amplified, the fluorescence signal was amplified further with the Evagreen added. **b** The cell mixture with the target MCF-7 cells illuminated by the THE-amplification system was imported into the FADS system, and the droplets encapsulated with the MCF-7 cells were selectively sorted

In this work, an ultra-sensitive single-cell sorting platform was developed by integrating FADS with a Tetramer-HCR-EvaGreen signal amplification system comprised of aptamer-connected tetrahedral DNA nanostructures (Apt-TDNs), HCR and EvaGreen dye (THE-FADS), as illustrated in Scheme 1. The Apt-TDNs was used to bind epithelial cell adhesion molecular (EpCAM) expressed on the membrane of MCF-7 cells. The extended DNA strands on the other three vertices of the Apt-TDNs acted as activatable initiators for HCR, triggering concomitant fluorescence signal amplification. The incorporated EvaGreen dye could amplify the fluorescent signal further. The THE-amplified MCF-7 cells in the mixture solution were imported into the FADS system, compartmentalized into individual droplets, and then selectively sorted. Subsequently, the sorted MCF-7 cells were recultured together with the CK19 and EGFR mRNA expression were analyzed.

## Materials and methods

### Materials and reagents

All DNA sequences were synthesized, labelled, and purified by Sangon Biotechnology Inc. (Shanghai, China), and the sequences were shown in Table S1. Phosphate buffer solution (PBS), TM buffer, tris (hydroxymethyl) aminomethane (Tris), TE buffer, TBE buffer, and magnesium chloride (MgCl_2_) were purchased from Sangon Biotechnology Inc. One-Step RT-qPCR Kit and total RNA Extractor (Trizol) were purchased from Sangon Biotechnology Inc. Optiprep was purchased from Sigma-Aldrich Company Ltd. The human breast cancer cell line (MCF-7) and human cervical cancer cell line (HeLa) were purchased from the Cell Bank of the Chinese Academy of Sciences (Shanghai, China). Dulbecco’s modified Eagle medium (DMEM; Gibco), Penicillin-streptomycin (PS), Fetal bovine serum (FBS; Gibco), 0.25% Trypsin-EDTA (1X), CellTracker™ Green CMFDA, CellTracker™ Orange CMFDA, Calcein (AM), Ethidium homodimer-1 (EthD-1), DAPI, and Hochest 33342 were purchased from Life Technologies. Alexa488-anti-EpCAM Rabbit monoclonal antibody (ab237395, Abcam) was obtained from Abcam Technology (Cambridge, UK). The SU-8-negative photoresist was purchased from MicroChem (MA, USA). Polydimethylsiloxane (Sylgard 184) and curing agent were obtained from Dow Corning (Shanghai, China). The film photomask was ordered from Gx Photomask Co., Ltd. (Shenzhen, China).

### Preparation and characterization of the THE system

Apt-TDNs were synthesized according to the following protocol. Briefly, the aptamer strand and four base strands (tetra-A, tetra-B, tetra-C, tetra-D) were separately dissolved in TE buffer (10 mM Tris, 1 mM EDTA, pH = 8.0) to a final concentration of 10 μM. Each DNA strand with the same volume (5 μL) was mixed with TM buffer (25 μL, 20 mM Tris, 50 mM MgCl2, pH = 8.0). The resulting mixture was heated using a BioRad T100 thermal cycler with 95 °C for 10 min, followed by cooling to 4 °C in 30 s. Apt-TDNs were identified by native polyacrylamide gel electrophoresis (PAGE). An 8% polyacrylamide gel solution (6 mL) was prepared with 30% acrylamide/bis-acrylamide solution (1.6 mL), MgCl_2_ (0.75 mL), 5 X TBE (1.2 mL), and Milli-Q water (2.45 mL). 60 μL ammonium persulfate (APS) and 6 μL 1,2-di- (dimethylamino) ethane (TEMED) were added into the polyacrylamide gel solution and mixed quickly for the preparation of PAGE gel. All the DNA samples were run at 100 V for 120 min. After electrophoresis, the gel was stained with GelRed for 15 min and visualized under UV illumination.

Apt-TDNs-dendrimers (HCR reaction-based dendritic structures) were synthesized based on the following protocol. Firstly, Substrate-A and Substrate-B were prepared separately by annealing mixtures of 3 μM F strand and 4.5 μM Q strand through a temperature cycle of 85 °C for 5 min followed by cooling to ambient temperature with the rate of 1 °C/s. Secondly, the annealed Substrates-A and Substrates-B were mixed with the corresponding 4.5 μM Assistants (Assistant-A and Assistant-B) separately and incubated for 20 min. Thirdly, these two resulting solutions were mixed with a ratio of 1:2 with the final concentrations of 0.5 μM Substrate-A, 0.75 μM Assistant-A, 1 μM Substrate-B, and 1.5 μM Assistant-B (H solution). Finally, Apt-TDNs were added into the H solution to form Apt-TDNs-dendrimers. The PAGE was employed to characterize the assembling of Apt-TDNs-dendrimers. Apt-TDNs at various concentrations (0.05, 0.1, 0.25, 0.5 μM) were mixed with the H solution to form Apt-TDNs-dendrimers with different sizes. Additionally, the H solution, 1 μM Apt-TDNs, 1 μM Substrate-A and 1 μM Substrate-B were also prepared for electrophoresis.

For atomic force microscopy (AFM) imaging of the Apt-TDNs-dendrimers, 10 μL 20 nM Apt-TDNs-dendrimers was diluted in TM buffer and subsequently filtered by a 0.22 μm membrane filter. The solution was then dropped in APTES pre-treated mica and adsorbed for 30 ~ 40 s. After this, the mica surface was rinsed four times with 50 μL water. Excess water was then removed using argon, and the mica was further dried under a vacuum for at least 20 min. AFM images were conducted using Dimension FastScan (Bruker, Germany). An identical protocol was applied to the AFM imaging of the Apt-TDNs.

For hydrodynamic size measurement of the Apt-TDNs-dendrimers, 200 μL 500 nM Apt-TDNs-dendrimers was diluted in water. 10 μL solution was carefully added into the bottom of a cuvette to avoid bubble formation. The dynamic light scattering (DLS) -based hydrodynamic size measurement was conducted by Zetasizer Nano ZS (Malvern, U.K.). An identical protocol was applied to measure the hydrodynamic size of the Apt-TDNs.

The fluorescence intensity of the THE signal amplification system was characterized by a microplate reader. Firstly, H solution, 1X EvaGreen solution (E solution), and the mixture solution of H solution and E solution (H-E solution) were prepared, respectively. Secondly, 0.1 μM Apt-TDNs solution was added into the H solution, H-E solution respectively to trigger the HCR reaction for signal amplification. Lastly, the fluorescence intensity of all samples was measured by a microplate reader (Synergy H1, BioTek) with excitation and emission wavelengths at 485 and 525 nm, respectively.

### Cell culture, imaging and fluorescence characterization

MCF-7 and Hela cells were cultured in the DMEM media supplemented with 10% FBS and 1% PS solution. All cells were maintained at 37 °C in a 5% CO_2_ atmosphere. The cells were collected using 0.25% trypsin-EDTA when cultivated to 80-90% confluence in cell culture dishes, and the cell number was determined using a hemocytometer.

To demonstrate the signal amplification ability of the THE system on MCF-7 cells, experiments were divided into three groups: control group, HCR group and HCR-Eva group. For all experimental groups, the MCF-7 cells were diluted into 2 × 10^5^ cells / mL. For the control group, the MCF-7 cells were centrifuged at 1000 rpm for 1 min, resuspended with 200 μL Apt-TDNs solution in binding buffer (5 mM MgCl_2_ in PBS, pH = 7.4). After 40 min of incubation on ice, the cells were washed with a binding buffer. Then the MCF-7 cells were divided into two groups, one was for imaging by confocal microscopy (FV3000, Olympus), the other was centrifuged at 1000 rpm for 1 min and resuspended with 200 μL H solution. After incubation of 37 °C for 40 min, the cells were washed with binding buffer again. Then the MCF-7 cells were divided into two groups, one was for confocal imaging, the other was centrifuged at 1000 rpm for 1 min, resuspended with 200 μL E solution. After incubation of 37 °C for 20 min, the cells were washed with binding buffer, then imaged by confocal microscopy.

For the fluorescence intensity quantification, 10^4^ MCF-7 cells from each experimental group were added into the wells of a microplate in 50 μL PBS solution. Fluorescence intensity was then measured with an excitation wavelength of 485 nm and an emission wavelength of 525 nm by a microplate reader.

To demonstrate the specific signal amplification ability of the THE system for MCF-7 cells, the Apt-TDNs solution, H solution and E solution were added into the MCF-7 cells or Hela cells solution separately, the following protocol for signal amplification of the THE system on MCF-7 cells was the same as above. Then the MCF-7 and Hela cells were imaged by confocal microscopy respectively.

For the cell viability characterization, the MCF-7 cells from all the experimental groups were stained with Calcein-AM/EthD-1 mixture (2 µM each) separately at 37 °C for 20 min. Then the cells were imaged by confocal microscopy. The viable and dead cells were counted using ImageJ software. Cell viability was calculated as the ratio of viable cells to the total number of cells.

The MCF-7 cells from all the experimental groups were also collected for reculture experiments. Briefly, the MCF-7 cells from each experimental group were seeded into a 24-well plate and cultured in DMEM media supplemented with 10% FBS and 1% PS solution at 37 °C in a 5% CO_2_. The MCF-7 cells were imaged in 0 days and 2 days by confocal microscopy.

### Microfluidic chip design and fabrication

The microfluidic chip was fabricated employing soft lithography techniques^44,45^as the following steps: SU-8 3025 photoresist (MicroChem) was spin-coated at 1000 rpm onto a 3-inch silicon wafer to achieve a depth of ~60 µm for the first layer. Following a pre-bake at 95 °C for 20 min, a photomask was positioned on the wafer, which was then exposed to 120 mW UV light (M365L2, Thorlabs) for 5 min. After a post-bake phase at 95 °C for 10 min, a second coating of SU-8 3025 photoresist was applied at 1000 rpm and baked for 45 min. Then a second photomask was used, and the wafer was exposed to UV light for 5 min. Following a final bake at 95 °C for 20 min, the wafer was developed in an SU-8 developer solution (MicroChem) for 15 min. The fabricated master was subsequently cleaned with isopropanol and ethanol, and then dried with a stream of nitrogen. A 10:1 (w:w) mixture of the PDMS precursor (SYLGARD 184, Dow Corning) and the curing agent was poured over the master, followed by an overnight curing process at 60 °C. The PDMS slab was detached from the master, and a 0.7 mm hole puncher was employed to fabricate inlet/outlet ports. Oxygen plasma treatment was used to bond the PDMS slab to a glass slide. The chip was baked at 100 °C for 30 min and 65 °C overnight to enhance this bonding strength. Lastly, the channels in the chip were wetted by Aquapel (PPG Industries) to render the channel surfaces hydrophobic. Then the chip was baked at 60 °C for 10 min before use.

### Construction and optimization of the FADS

The designed FADS microfluidic chip integrated a primary fluidic component and an auxiliary component. The primary fluidic component comprised three inlets (cell, spacer, bias) and two outlets (waste, sorted). The cell inlet was used to input the cell solution. The spacer inlet and bias inlet were injected with a fluorinated oil containing 2% (v/v) PEG-PFPE surfactant (oil phase) separately. The waste outlet was connected to a suck pump and was used to collect the undesired droplets (empty droplets or droplets with un-target cells). The sorted outlet was used to collect the desired droplets (droplets with target cells). The auxiliary component comprised a moat, an electrode, and two optical fibers. The electrode and moat were injected with 4 M KCl solution. Two optical fiber channels were inserted with laser optical fiber and a PMT optical fiber separately. The electrode and moat were both filled with 4 M KCl solution. The dye in the droplet was excited by laser fiber with a 473 nm laser, and a PMT fiber was used to detect the fluorescence signal in real time. The FADS microfluidic chip was placed on a sorting machine (CytoSpark droplet sorting system, Zhejiang Dapu Biotechnology Co., Ltd).

To evaluate the practicability of the FADS system, the experiments were classified into control group, antibody group, HCR group and HCR-Eva group. For the antibody group, the MCF-7 cells were incubated with 5 μg/μL EpCAM antibody for 40 min on ice, then the cells were washed with binding buffer. For the other three groups, the signal amplification of the THE system on MCF-7 cells followed the protocol in “cell culture, imaging and fluorescence characterization” part. The MCF-7 Cells from each experimental group were separately introduced into the FADS microfluidic chip. The flow rate of cell inlet, spacer oil inlet, bias oil inlet and waste outlet were set as 100 μL/h, 400 μL/h, 500 μL/h and - 500 μL/h, respectively. After the flow rate was stable, the signals of droplets across each group were collected.

### MCF-7 cells sorting by the THE-FADS in PBS solution

MCF-7 cells and Hela cells were stained with 5 µg/mL DAPI and 5 µM Cell Tracker Orange for 15 min respectively, then mixed together with the ratio of 1:1. After that, the Apt-TDNs solution, H solution and E solution were added into the cell mixture sequentially. The signal amplification of the THE system on MCF-7 cells in the cell mixture followed the protocol in “cell culture, imaging and fluorescence characterization” part. Then the cell mixture was centrifuged at 1000 rpm for 1 min. After the supernatant was discarded. The cell mixture was resuspended in OptiPrep-PBS (20% OptiPrep in PBS with 0.02% BSA) and loaded into a 1 ml syringe connected to the cell inlet of the FADS microfluidic chip with the flow rate of 100 μL/h. The spacer inlet and bias inlet were filled with HFE-7500 fluorinated oil containing 2% (v/v) PEG-PFPE surfactant by a 2 mL syringe, with a flow rate of 400 μL/h and 500 μL/h, respectively. The waste outlet was constantly sucked by a connected 2 mL syringe with the flow rate of - 500 μL/h. A 2 V PMT voltage threshold was set to perform sorting. The sorted droplets with MCF-7 cells were collected in a 1.5 mL Eppendorf tube and imaged by confocal microscopy.

To test the sorting purity of the THE-FADS system, the MCF-7 cells were diluted into varying numbers (20, 200, 1000, 2000) in a 200 μL OptiPrep-PBS solution, the Hela cells were prepared with the concentration of 2 × 10^5^ cells / mL. The protocol of MCF-7 cells and Hela cells for staining, mixing together with signal amplification of the THE system on MCF-7 cells was the same as above except that the MCF-7 cells with varying numbers mixed with Hela cells. The sorting purity was defined as the ratio of sorted MCF-7 cells to the total number of sorted cells.

To test the sorting efficiency of the THE-FADS system, the MCF-7 cells were diluted into varying numbers (20, 200, 1000, 2000) in a 200 μL OptiPrep-PBS solution. After that, the Apt-TDNs solution, H solution and E solution were added into the MCF-7 cells sequentially. The signal amplification process of THE system on MCF-7 cells followed the protocol in “cell culture, imaging and fluorescence characterization” part. Then the MCF-7 cell solution was injected into the FADS microfluidic chip for sorting. The sorting efficiency was defined as the ratio of sorted MCF-7 cells to the total number of spiked MCF-7 cells.

For FACS-based MCF-7 cells sorting, the MCF-7 and Hela cells were mixed with the ratio of 1:100 and incubated with 5 μg/μL EpCAM antibody for 40 min on ice. Then the cell mixture was washed with binding buffer. The cell mixture was imported into a flow cytometer (FACS Aria III, BD Biosciences) to sort MCF-7 cells.

To compare the cell viability of MCF-7 cells obtained by the THE-FADS system and the antibody-based FACS system, 4000 MCF-7 cells from each sorting system were recultured. After three days, the MCF-7 cells were imaged by confocal microscopy.

### MCF-7 cells sorting by the THE-FADS in the mimic serum samples and downstream analysis

The staining and mixing of MCF-7 cells and Hela cells, signal amplification of MCF-7 cells by the THE system was the same as that in PBS solution except that the ratio of MCF-7 cells and Hela cells in mixture solution was 1:100. The sorting procedure was the same as that in PBS solution except that OptiPrep-FBS (20% OptiPrep in FBS) was used to replace PBS. After obtaining the MCF-7 cell-laden droplets, the cells were recovered by adding 100 µL DMEM media supplemented with 10% FBS, followed by 100 µl 1H,1H,2H,2H-perfluoro-1-octanol. The mixture was then gently mixed and centrifuged at 700 *g* for 1 min to favor complete phase separation. The bottom oil was discarded, and the MCF-7 cells were obtained.

For downstream molecular analysis, the MCF-7 cells sorted by the THE-FADS in mimic serum samples were recultured and imaged by confocal microscopy. After the MCF-7 cells were cultivated to 80–90% confluence, the cells were digested with trypsin. Total RNA was extracted from the recultured MCF-7 cells using Trizol according to the manufacturer’s instructions. The normal cultured MCF-7 cells were set as the control. Extracted RNA was used in a one-step RT-qPCR reaction with Taq DNA Polymerase to amplify CK19 and EGFR genes. The PCR procedures were as follows: 50 °C for 5 min, 95 °C for 3 min, 40 cycles of 95 °C for 10 s and 60 °C for 30 s.

## Results and discussion

### Principle and characterization of the THE signal amplification system

**Fig. 1:**
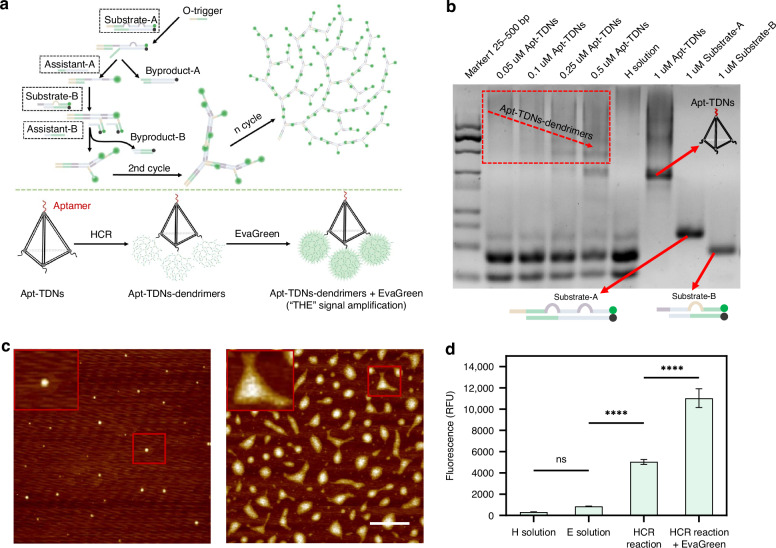
Construction and characterization of the THE signal amplification system. **a** The schematic of the HCR isothermal amplification. A dendritic DNA nanostructure was constructed by O-trigger strand with Substrate-A, Substrate-B, Assistant-A and Assistant-B. The Apt-TDNs-dendrimers would be formed by initiating HCR on three vertexes of Apt-TDNs. The THE signal amplification system would be constructed by introducing EvaGreen into the Apt-TNDs-dendrimers. **b** PAGE characterization of the Apt-TDNs-dendrimers. Lane1, 25–500 bp marker; Lane2-5, Apt-TDNs-dendrimers formed when Apt-TDNs solution with different concentration (0.05, 0.1, 0.25, 0.5 μM) were mixed with H-solution; lane6, H solution; lane7, 1 uM Apt-TDNs solution; lane8-9, two HCR substrate, Substrate-A and Substrate-B solutions. **c** The AFM characterization of the Apt-TDNs and Apt-TDNs-dendrimers. Left: Apt-TDNs, right: Apt-TDNs-dendrimers. The scale bar is 1000 nm. **d** Bar chart of fluorescence intensity quantification by microplate reader for H solution, E solution, HCR and HCR plus EvaGreen. The error bars represented the standard deviation of three measurements. All measurements were performed in triplicate, and error bars represented the ± SD. ****P < 0.0001, Student’s *t*-test

The mechanism of Apt-TDNs-based HCR amplification was shown in Fig. 1a. The HCR substrate comprised two kinds of substrates (Substrate-A, Substrate-B) and two kinds of assistant strands (Assistant-A, Assistant-B). Each substrate comprised a quencher-labelled DNA strand (Q-strand) and a fluorophore-labelled DNA strand (F-strand), with the Q-strand sequence complementary to the F-strand. Upon Q-F double-helix formation, the fluorophore labelled in F-strand was quenched (off). There are two loops in the formed substrate A and one loop in the formed substrate B. Assistant-A and Assistant-B were designed to be complementary to part of the Q strand sequence of Substrate-A and Substrate-B, respectively. The trigger (O-trigger) was a single-strand DNA sequence complementary to the toehold of Substrate-A. Once the O-triggers solution was mixed with the prepared HCR substrate solution, it would firstly hybridize with the exposed toehold of Substrate A, displaced part of the Q strand, exposing a new toehold and opened the first loop. Assistant-A then interacted with the Q-strand in Substrate-A, forming a double helix structure that released the previously quenched fluorophore-labelled in the F-strand, generating a fluorescence signal (on). Subsequently, two identical sequences within the F-strand of Substrate-A concurrently hybridized with the toeholds of Substrate-B, displaced part of the Q strand, exposing a new toehold and opening the loop in Substrate-B. The Assistant-B would interact with the Q-strand in Substrate-B, forming a double helix structure that released the previously quenched fluorophore labelled in the F-strand. The fluorescence signal was generated (on). Following the formation of the Q strand and Assistant strand double helix structure, more fluorophores were released. The newly exposed trigger sequences would initiate the subsequent reactions. When Apt-TDNs triggers (A-triggers) were introduced, it would initiate the HCR amplification, and three dendritic structures on the three vertexes of Apt-TDNs (Apt-TDNs-dendrimers) were formed. The release of Q strand in substrate A and substrate B would cause fluorescence production, which augmented the signal for the first time. The EvaGreen was utilized to amplify the signal for the second time. EvaGreen composed of two acridine orange moieties by a flexible linker, could exhibit weak fluorescence in a “closed form” without dsDNA; while upon the appearance of dsDNA, the acridine orange moieties dissociated and intercalated into the dsDNA, resulting in high fluorescence in an “opened form”^40,41^. The reason that EvaGreen dyes could augment the signal was that they could bind the dsDNA of Apt-TDNs-dendrimers and release the fluorescence signal.

The Apt-TDNs were composed of four single DNA oligonucleotide strands and one aptamer strand (Fig. S1). The single strand (a), double-strand combination (ab), triple-strand combination (abc), four-strand combination (abcd) and Apt-TDNs (abcd-apt) were synthesized and characterized through PAGE. The results showed that the migration rate of the samples a, ab, abc, abcd, and abcd-apt (Apt-TDNs) decreased gradually, corresponding to the gradual increase of the molecular weight (Fig. S2). The Apt-TDNs exhibited the slowest migration rate with the highest molecular weight, thereby confirming its successful formation. Apt-TDNs solution with various concentrations were added to H solution to initiate the HCR for the synthesis of Apt-TDNs-dendrimers. The characterization of the synthesized Apt-TDNs-dendrimers, as well as H solution, 1 μM Apt-TDNs, 1 μM Substrate-A, 1 μM Substrate-B, were conducted through PAGE (Fig. 1b). The experimental results demonstrated that with the increase of the Apt-TDNs concentration, the migration rate of the formed Apt-TDNs-dendrimers increased gradually, corresponding to the gradual decrease of the molecular weight. The reason was that Apt-TDNs with higher concentration would lead to more HCR due to the limited availability of the H solution, hence producing smaller-sized Apt-TDNs-dendrimers. However, for the H solution, there were two bands corresponding to the Substrate-A, Substrate-B. As for 1 μM Apt-TDNs, Substrate-A and Substrate-B, a single band was displayed for all of them, and the molecular weight was consistent with that of the designed sequences.

The morphology of Apt-TDNs and Apt-TDNs-dendrimers was further analyzed using AFM. The AFM image of Apt-TDNs displayed a small tetrahedron structure (Fig. 1c, left). However, Apt-TDNs-dendrimers exhibited a substantial increase of size compared with the size of the Apt-TDNs. Moreover, the diverse size distribution proved dendritic structures forming at the three vertices of Apt-TDNs (Fig. 1c, right).

The DLS was utilized to characterize the hydrodynamic sizes of Apt-TDNs and Apt-TDNs-dendrimers (Fig. S3). The mean diameters of Apt-TDNs and Apt-TDNs-dendrimers were 24 nm and 210 nm, respectively, further validating the successful construction of Apt-TDNs and Apt-TDNs-dendrimers.

Following the construction and characterization of Apt-TDNs and Apt-TDNs-dendrimers, we proceeded to characterize the THE signal amplification system through fluorescence intensity quantification. It was shown (Fig. 1d) that the mean relative fluorescence units (RFU) of the H solution were 322 due to the insufficient quenching of the fluorophore on both Substrate-A and Substrate-B. The mean RFU of E solution was 860 due to the background signals of acridine orange moieties. The mean RFU of HCR-formed Apt-TDNs-dendrimers was 5043 due to the release of the Q-strand from both Substrate-A and Substrate-B. For the HCR and EvaGreen amplification system, the fluorescence signal could be enhanced further, reaching an RFU of 1,1036, which was thirty-four times higher than that of the H solution and twelve times higher than that of E solution. These results clearly indicated that the THE signal amplification system exhibited the most pronounced signal amplification effect.

### Cell fluorescence signal amplification by the THE signal amplification system

The feasibility of the THE signal amplification system at the cellular level was investigated. The aptamer SYL3C which could specifically recognize the EpCAM protein on the membrane of MCF-7 cells was used^46^.

**Fig. 2:**
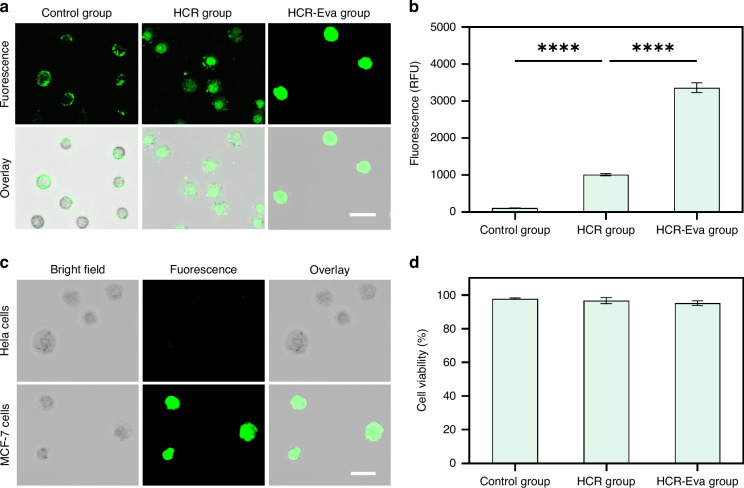
Characteristic of the THE signal amplification-based cells. **a** Confocal fluorescence images of the target MCF cells for the control group, HCR group and HCR-Eva group. The scale bar is 30 μm. **b** Bar chart of MCF-7 cells fluorescence intensity quantification for control group, HCR group and HCR-Eva group. The error bars represent that standard deviation of three measurements. **c** Confocal fluorescence images of the target MCF cells and non-target Hela cells incubated with 1 μM Apt-TDNs. The scale bar is 30 μm. **d** Bar chart of MCF-7 cells viability analysis in control group, HCR group and HCR-Eva group. All measurements were performed in triplicate, and error bars represented the ± SD. ****P < 0.0001, Student’s *t*-test

Confocal fluorescence microscopy was employed to evaluate the fluorescence signal intensity. It was shown that for the control group, a weak fluorescence signal was observed. For the HCR group, a notable fluorescence intensity increased. For the HCR-Eva group, a substantial enhancement in fluorescence intensity on the MCF-7 cells was observed, which was higher than the HCR group (Fig. 2a). The high signal was attributed to the interaction between EvaGreen and Apt-TDNs-dendrimers, leading to the release of a high-intensity fluorescence signal. What’s more, the different cell types with different EpCAM protein expression would have different signal intensity, which was demonstrated by several previous studies^47,48^.

The microplate reader was used to quantify the fluorescence intensity. For the control group, the fluorescence intensity was 109. For the HCR group, the fluorescence intensity increased to 1015. For the HCR-Eva group, a fluorescence intensity with the value of 3362 was observed, which was thirty-times higher than that of the control group (Fig. 2b). These results further confirmed that THE signal amplification system exhibited the highest level of signal amplification effect.

The MCF-7 and Hela cells after THE signal amplification were used to assess the specificity. The results demonstrated that the fluorescence signal was specifically localized on MCF-7 cells with high EpCAM expression. However, no fluorescence signal was observed on the Hela cell without EpCAM expression (Fig. 2c). These results proved that the THE signal amplification system exhibited excellent specificity towards MCF-7 cells.

To evaluate the cell viability, the MCF-7 cells were stained with Calcein-AM/EthD-1 (Fig. S4). The Calcein-AM (green) was used to stain the viable cells, while EthD-1 (red) was used to stain the dead cells. The results revealed that most of the cells exhibited high viability following the treatments and more than 95% cell viability was obtained for all experimental groups (Fig. 2d). To further validate the cell viability, a reculture experiment was performed. The results showed that the cells from all experimental groups were propagated well after a two-day culture (Fig. S5), which provided further evidence of high cell viability.

### Construction and optimization of the FADS

**Fig. 3:**
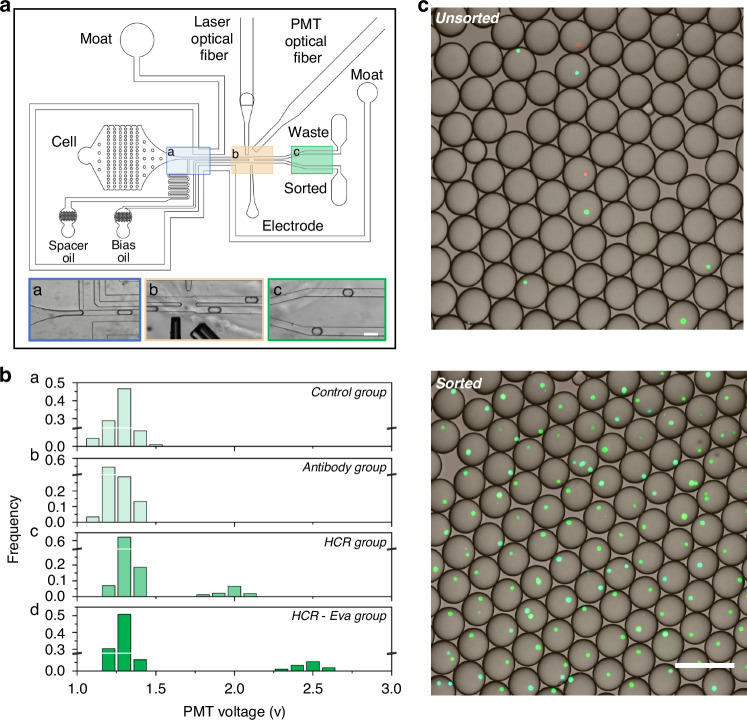
Construction of the THE-FADS. **a** Design of the microfluidic chip used for the THE-FADS. The three inlets on the bottom shows the region of droplet making (a, blue), droplet sorting (b, yellow) and droplet collection (c, green). The scale bar is 200 μm. **b** Distribution of peak signal intensity across various group. From top to bottom, the rows a, b, c and d represent the control group, antibody group, HCR group, and HCR-Eva group, respectively. **c** Confocal fluorescence images of the obtained droplets under unsorting and sorting mode, respectively. When no sorting voltage is applied, a few droplets have MCF-7 cells (upper). When sorting voltage is applied, most of the droplets have MCF-7 cells (lower). The scale bar is 200 μm

Here, we designed a microfluidic chip that comprised a primary fluidic component and an auxiliary component to construct and optimize the FADS. The primary fluidic component has three inlets (cell, spacer, bias) and two outlets (waste, sorted), featuring a droplet generation junction (Fig. 3a, blue rectangle), droplet sorting junction (Fig. 3a, yellow rectangle), and droplet collection region (Fig. 3a, green rectangle). The structure was specifically designed for the generation, sorting, and collection of cell-laden droplets. During the sorting of MCF-7 cells, the cell inlet was infused with cell suspension containing the MCF-7 cells. The oil phase in the spacer inlet and bias inlet were used to facilitate droplet generation and fluid stabilization. The sorted outlet was for the collection of selected droplets, while the waste outlet was for empty droplets and non-target cell-laden droplets.

The auxiliary component integrated a moat, an electrode, and two optical fibers. The moat, filled with 4 M KCl solution, enveloped the primary fluidic component, acting as a barrier against stray electric fields that could unintentionally induce droplet coalescence. The electrode, also infused with 4 M KCl solution, operates in unsorting mode and sorting mode. In the unsorting mode, the electrode remained unpowered, thus no sorting happened. Conversely, during the sorting mode, the electrode became powered, and produced a DEP force to the MCF-7 cell-laden droplets, thereby these droplets were effectively sorted. The optical fibers, consisting of a laser fiber and a PMT fiber, could supply excitation light and collect emission light, respectively.

When used for MCF-7 cells sorting, a cell suspension containing MCF-7 cells was introduced via the cell inlet and subsequently encapsulated by the spacer oil at the droplet generation junction, yielding cell-laden droplets. These droplets flowed to the sorting junction and encountered the bias oil. Each cell-laden droplet was illuminated by a focused laser beam, the fluorescent dyes on the cells were excited and subsequently emitted fluorescence was generated.

To evaluate the practicability of our FADS system, signals of each group were captured by PMT fiber and transported into a computer; then they were analyzed by LabVIEW and FPGA, resulting in a PMT voltage output. The peak signal intensities of each group were utilized to yield a distribution graph with the horizontal axis as PMT voltage and the vertical axis as the frequency of droplets (Fig. 3b).

For the control group, the MCF-7 cell-laden droplets had a similar signal intensity with the empty droplets, proving that the cell-laden droplets cannot be separated by the FADS system. For the antibody group, the MCF-7 cell-laden droplets displayed similar signal intensities with empty droplets, indicating their inability to be distinguished by the FADS system. For the HCR group, the signal intensity of the MCF-7 cell-laden droplets and empty droplets exhibited two distinct populations, indicating that MCF-7 cell-laden droplets could be separated by the FADS system. For the HCR-Eva group, the signal intensity of the MCF-7 cell-laden droplets and empty droplets exhibited two markedly separated populations, suggesting these MCF-7 cell-laden droplets were easily recognized by the FADS system (Fig. 3b). These results demonstrated that the HCR-Eva group could produce the most pronounced signal amplification, and these MCF-7 cell-laden droplets had the best discrimination from empty droplets.

To further establish a useful FADS system, we proceeded to establish an optimal threshold voltage specifically for the sorting of MCF-7 cell-laden droplets. Cells from the HCR-Eva group were employed and a PMT threshold of 2 V was established (Fig. S6). When the droplet signal exceeded this threshold, it was designated as positive. Conversely, signals below the threshold were classified as negative.

### Construction of the THE-FADS and MCF-7 cell sorting in PBS solution

Following the successful establishment of the THE signal amplification and FADS systems, we endeavored to synergize these methodologies to construct the THE-FADS system. To facilitate subsequent cell tracking, the MCF-7 cells were stained with DAPI (blue) and Hela cells with Cell Tracker Orange (red), respecitively. The cell mixture was introduced into the FADS microfluidic chip for the sorting of MCF-7 cell-laden droplets. In the unsorting mode, all droplets, regardless of their contents, flowed directly to the waste outlet. The collected droplet populations in this mode comprised MCF-7 cell-laden droplets, Hela cell-laden droplets, and empty droplets (Fig. 3c, upper). Conversely, in the sorting mode, the dye in the MCF-7 cell-laden droplets was activated and the fluorescence signal was collected by the PMT fiber, then the MCF-7 cell-laden droplets were selectively sorted to the sorted outlet by DEP force, while Hela cell-laden droplets and empty droplets flowed towards the waste outlet (Fig. 3c, lower).

To evaluate the sorting purity of the THE-FADS system, the MCF-7 cells with different numbers were spiked into a OptiPrep-PBS solution containing Hela cells. The sorted cell-laden droplets were imaged and enumerated by a confocal microscopy. Remarkably, an average purity of 91% was achieved for all spiked cell numbers (Fig. S7). The results indicated that a high purity of MCF-7 cells could be obtained by THE-FADS, which was identical to other FADS technologies^16,49,50^. What’s more, the stability of the Apt-TDNs on the cell surface was demonstrated by the no fluorescence leakage, as well as the several previous studies^48,51^.

To ascertain the sorting efficiency of the THE-FADS system, the MCF-7 cells with different numbers were spiked into a OptiPrep-PBS solution. A strong linear relationship was observed between the number of spiked MCF-7 cells and the number of sorted MCF-7 cells by the THE-FADS. The linear regression equation was formulated as Y = 1.1681 X – 0.7034, with an R² value of 0.9992. With varying numbers of spiked MCF-7 cells, the THE-FADS system exhibited an average sorting efficiency of 55.5% (Fig. S8), which demonstrated the target cell enrich capability of THE-FADS.

To further affirm the THE-FADS sorting strategy had minimal damage, we compared the cell viability by the THE-FADS system and the antibody-based FACS system. After three days, a small cell population was formed for the THE-FADS-obtained MCF-7 cells (Fig. S9, right). The cultured cells exhibited high cell viability, intact cell morphology and potent proliferation capability, demonstrating a similar living state with that of the normally cultured cells. However, the MCF-7 cells obtained by the FACS system were scattered in a culture dish, without MCF-7 cells population being observed, indicating that the cells obtained by the FACS system had low cell viability (Fig. S9, left). What’s more, THE-FADS shows significant sorting marker difference and innovative signal amplification principle^13,14,16,17,52^, as shown in the Table S2.

### MCF-7 cell sorting in mimic serum samples by THE-FADS and downstream analysis

**Fig. 4:**
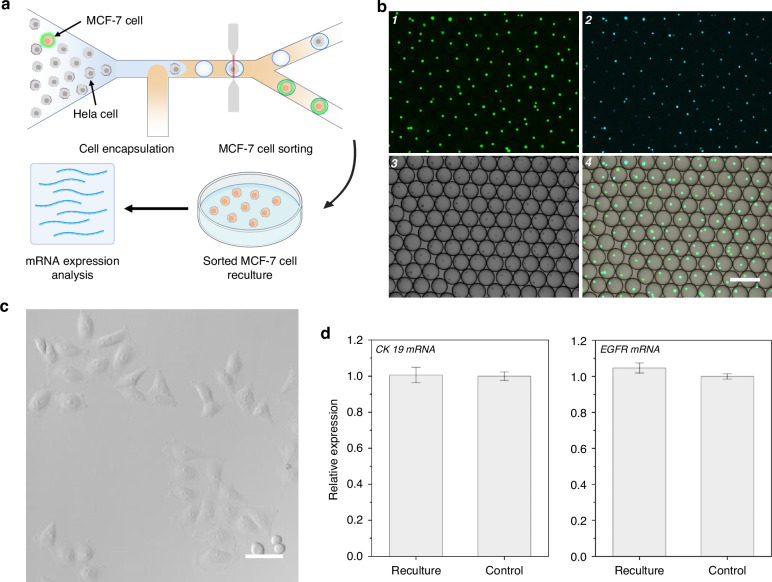
MCF-7 cell sorting by the THE-FADS in mimic serum sample and downstream analysis. **a** The schematic diagram of the experiment design. **b** Confocal fluorescence images of the sorted droplets containing MCF-7 cells from mimic serum sample. From 1 to 4: green fluorescence channel image, blue fluorescence channel image, bright field image and merge image. The scale bar is 200 μm. **c** Confocal images of the recultured MCF-7 cells obtained from THE-FADS in a mimic serum sample after 3 days culture. The scale bar is 50 μm. **d** Bar chart of EGFR mRNA and CK mRNA expression profile for recultured THE-FADS obtained MCF-7 cells (left) and control MCF-7 cells (right). All measurements were performed in triplicate, and error bars represented the ± SD

To better evaluate the performance of the THE-FADS method under more physiologically relevant conditions, we proceeded to sort MCF-7 cells from the mixture of MCF-7 cells and HeLa cells in a mimic serum sample. The sorted cells were recultured, and the expression of key genes CK19 (cytokeratin 19) and EGFR (epidermal growth factor receptor) were examined (Fig. 4a). The results showed that MCF-7 cells with high sort purity of 85% were obtained with an average sort efficiency of 50.3% in simulated serum samples (Fig. 4b), confirming that the sorting capability of the THE-FADS remained uncompromised under mimic serum conditions.

To prove the minimal detrimental impact of THE-FADS in a mimic serum sample, we recultured the obtained MCF-7 cells. After three days, the MCF-7 cells exhibited good adhesion properties, intact structures and high survival rates in the petri dish (Fig. 4c) which demonstrated that the MCF-7 cells could maintain high viability with THE-FADS treatment.

To assess the feasibility of the sorted cells for downstream molecular analysis, the expression of two MCF-7 cells-associated genes, CK19 and EGFR of the MCF-7 cells isolated from the THE-FADS were compared with that of the normal cultured MCF-7 cells (control). Upon direct lysis of these MCF-7 cells, CK19 and EGFR mRNA were detected using reverse transcription PCR (RT-PCR). The results revealed that the expression levels of EGFR mRNA and CK19 mRNA in the MCF-7 cells obtained via the THE-FADS were similar to that of the normal cultured MCF-7 cells (Fig. 4d). This suggested that the MCF-7 cells obtained through the THE-FADS technique did not affect the normal gene expression.

## Conclusions

In conclusion, we have developed an ultra-sensitive single-cell sorting platform by combining the FADS technology and the THE signal amplification system integrating Apt-TDNs, HCR and EvaGreen dye. The results showed that the fluorescence signal of the THE fluorescence amplification system increased significantly than that of the single Tetramer or Tetramer-HCR-based signal amplification system. Upon applied to MCF-7 cells, the platform demonstrated potent signal amplification on the cell surface, while maintaining high selectivity and more than 95% cell viability. Incorporating the THE-based fluorescence signal amplification technology into FADS resulted in a sorting efficiency of 55.5% and purity of 91% in PBS solution, with sorting efficiency of 50.3% and purity of 85% in simulated serum samples. The THE-FADS-sorted MCF-7 cells exhibited normal proliferation together with similar CK19 and EGFR mRNA expression compared to unsorted controls, confirming their adaptability for subsequent analytical steps. Taken together, the THE-FADS enhanced the capabilities of traditional FADS technology and offered exciting prospects for single-cell sorting and analysis, opening up vast opportunities for advancements in biomedical research, personalized medicine, and understanding of cellular heterogeneity.

## Supplementary information

Ultra-sensitive Fluorescence-Activated Droplet Single-cell Sorting based on Tetramer-HCR-EvaGreen Amplification
