# Supplementary material for: Ultra-sensitive fluorescence-activated droplet single-cell sorting based on Tetramer-HCR-EvaGreen amplification
Source: Microsyst Nanoeng. 2025 Jan 16;11:10. doi: 10.1038/s41378-024-00861-8 (PMC11739583; doi:10.1038/s41378-024-00861-8)
Supplement: Supplementary file 1 — Ultra-sensitive Fluorescence-Activated Droplet Single-cell Sorting based on Tetramer-HCR-EvaGreen Amplification [file 41378_2024_861_MOESM1_ESM.docx]

Supporting Information

Ultra-sensitive Fluorescence-Activated Droplet Single-cell Sorting based on Tetramer-HCR-EvaGreen Amplification

Long Chen ^a,b,d,1^, Yi Xu ^a,b,1^, Lele Zhou ^c^, Ding Ma ^a,d^, Rong Zhang ^c^ Yifan Liu ^c,f,g,^*, Xianqiang Mi ^a,b,d,e,^*

*^a^ National Key Laboratory of Materials for Integrated Circuits, Shanghai Institute of Microsystem and Information Technology, Chinese Academy of Sciences, Shanghai 200050, China*

*^b^ Shanghai Advanced Research Institute, Chinese Academy of Sciences, Shanghai 201210, China*

*^c^ School of Physical Science and Technology, ShanghaiTech University, Shanghai 201210, China*

*^d^ University of Chinese Academy of Sciences, Beijing 100049, China*

*^e^ School of Physics and Optoelectronic Engineering Hangzhou Institute for Advanced Study, University of Chinese Academy of Sciences, Chinese Academy of Sciences, Hangzhou, 310024, China*

*^f^ Shanghai Clinical Research and Trial Center, Shanghai 201210, China*

*^g^ State Key Laboratory of Advanced Medical Materials and Devices, ShanghaiTech University, Shanghai 201210, China*

**Corresponding author*

*Email address:* [*mixq@mail.sim.ac.cn*](mixq@mail.sim.ac.cn)*；*[*liuyf6@shanghaitech.edu.cn*](mailto:liuyf6@shanghaitech.edu.cn)

Supporting Figures and Tables

**Figure S1.** The schematic of Aptamer-connected tetrahedral DNA nanostructures (Apt-TDNs).


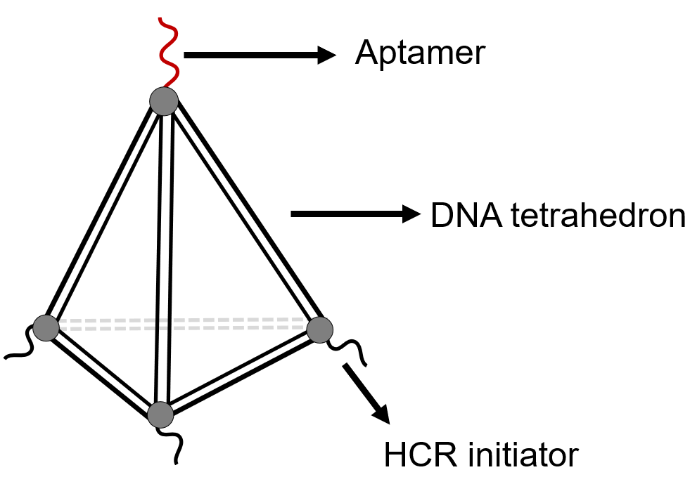


**Figure S2.** PAGE characterization of the Apt-TDNs. Lane 1, 25-500 bp marker; Lane 2-6, single strand (a), double-strand combination (ab), triple-strand combination (abc), four-strand combination (abcd) and Apt-TDNs (abcd-apt); Lane 7, 100-2000 bp marker.


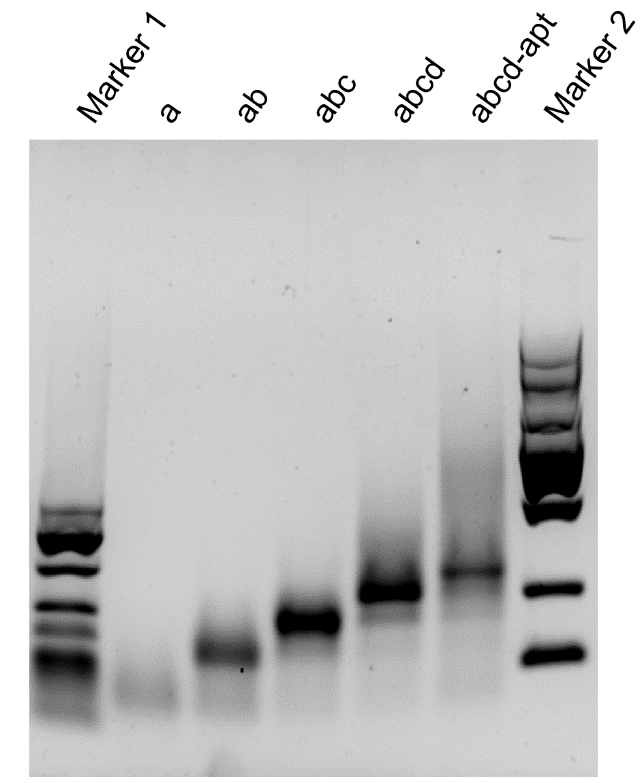


**Figure S5.** Confocal images of the MCF-7 cells after a two-day reculture for control group, HCR group and HCR-Eva group. The scale bar is 50 μm.


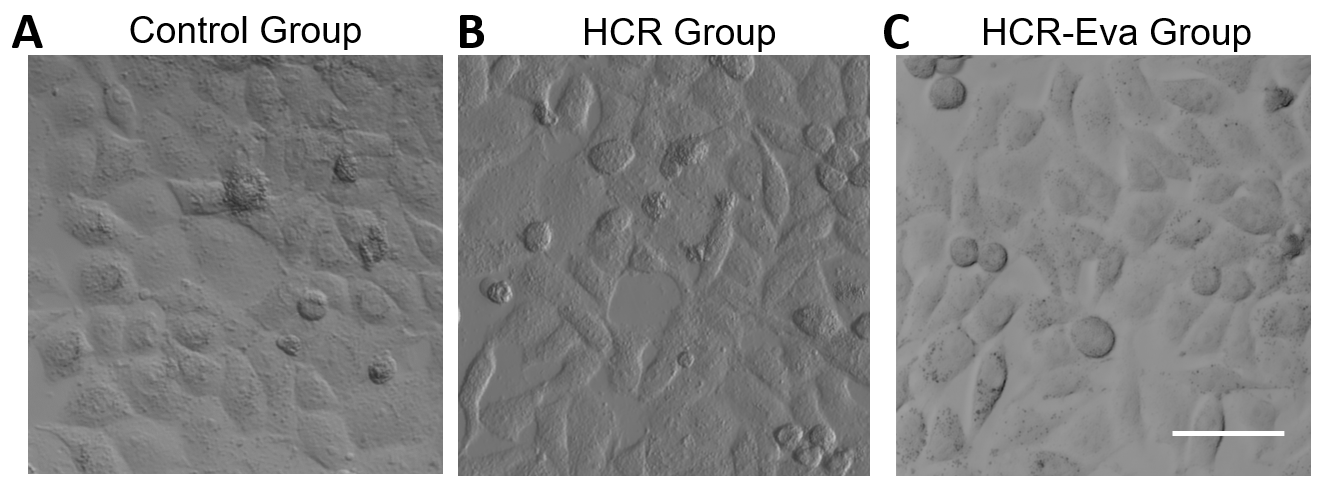


**Figure S4.** Confocal fluorescence images of the MCF cells after live/dead staining for control group, HCR group and HCR-Eva group. The scale bar is 100 μm.


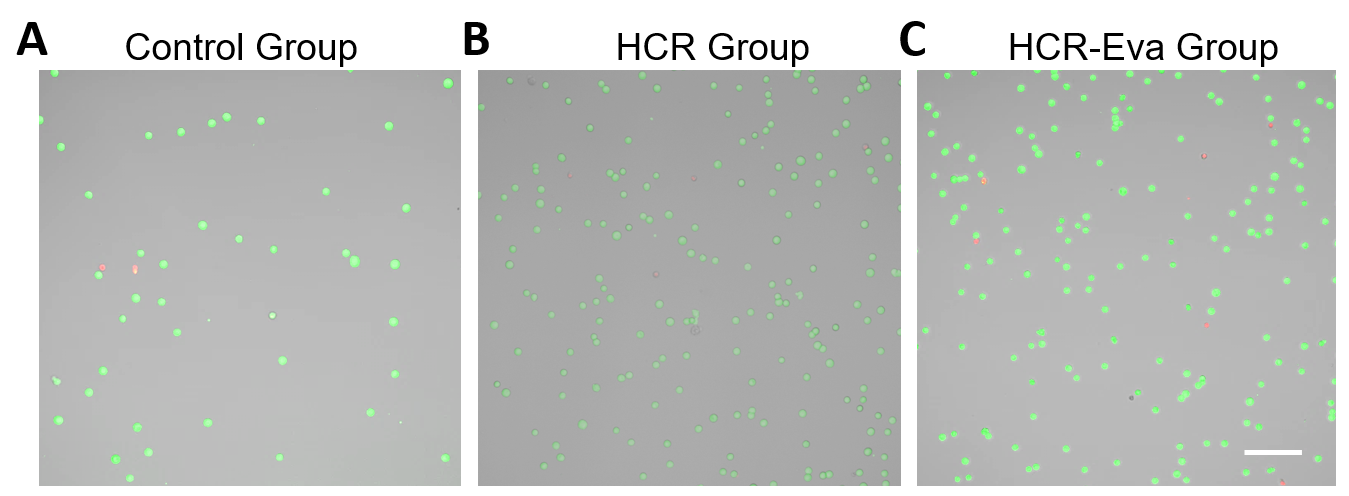


**Figure S3.** Hydrodynamic particle size distribution of (A) Apt-TDNs and (B) Apt-TDNs-dendrimers.


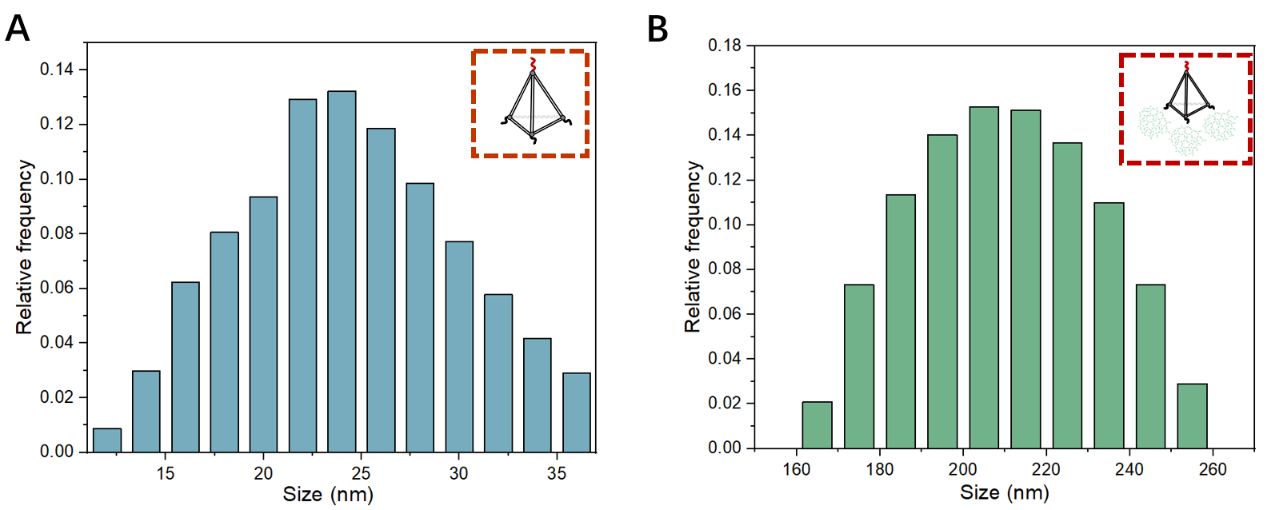


**Figure S7.** The sorting purity of the MCF-7 cells acquired by THE-FADS when MCF-7 cells with varying numbers (20, 200, 1000, 2000) were spiked into the Hela cells.


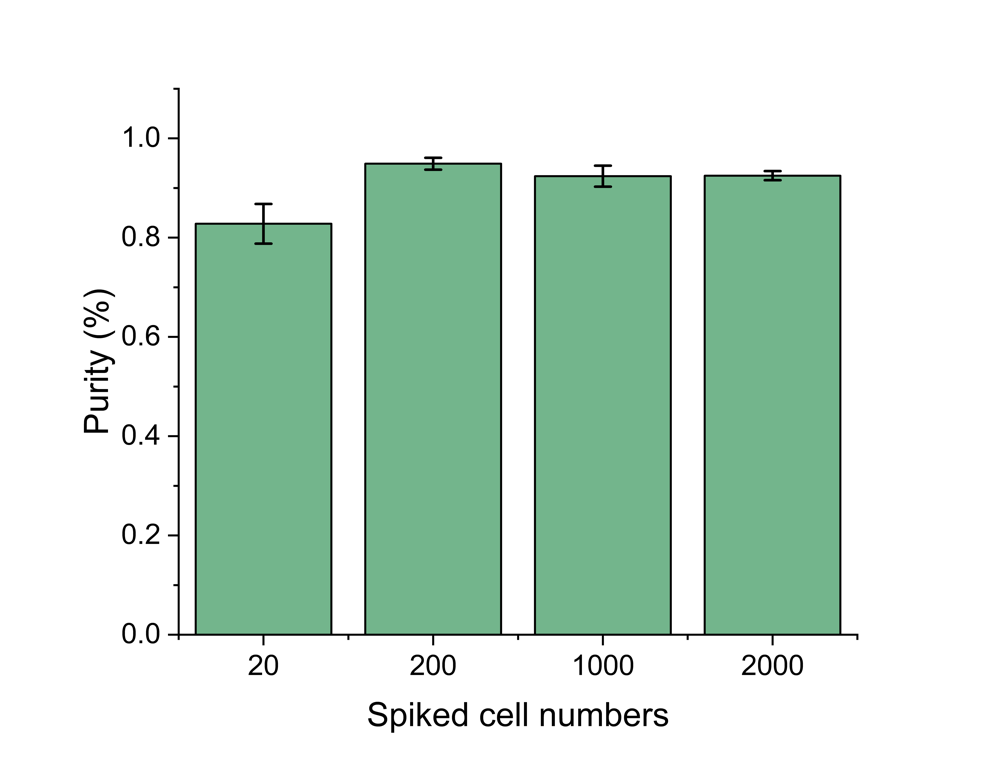


Figure S6. The PMT timetrace of recorded signals from the FADS system. The positive droplet shows a higher PMT voltage, while the negative droplet shows a lower PMT voltage. The positive droplet and negative droplet can be separated by setting an appropriate sorting threshold.


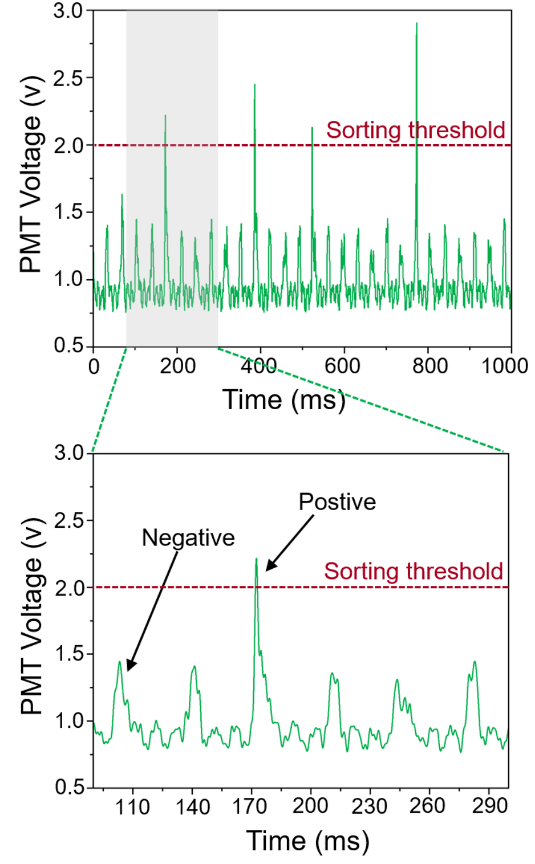


**Figure. S9.** Confocal images of the MCF-7 cells obtained from FACS (A, left) and THE-FADS (B, right) in PBS solution after three-day reculture. The scale bar is 50 μm.


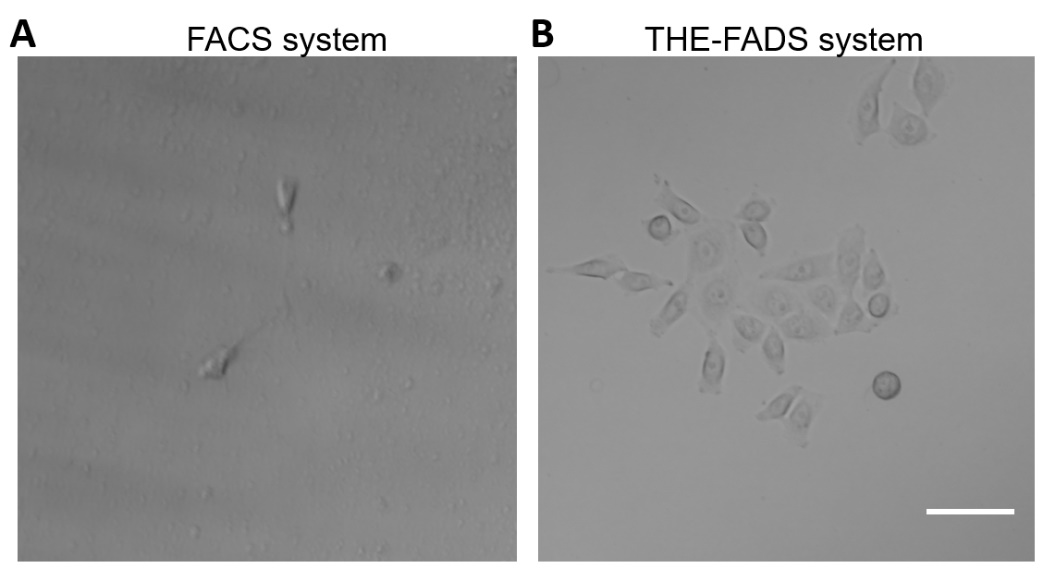


**Figure S8.** Regression analysis of the THE-FADS sorted MCF-7 cell number versus the spiked MCF-7 cell number in PBS solution.


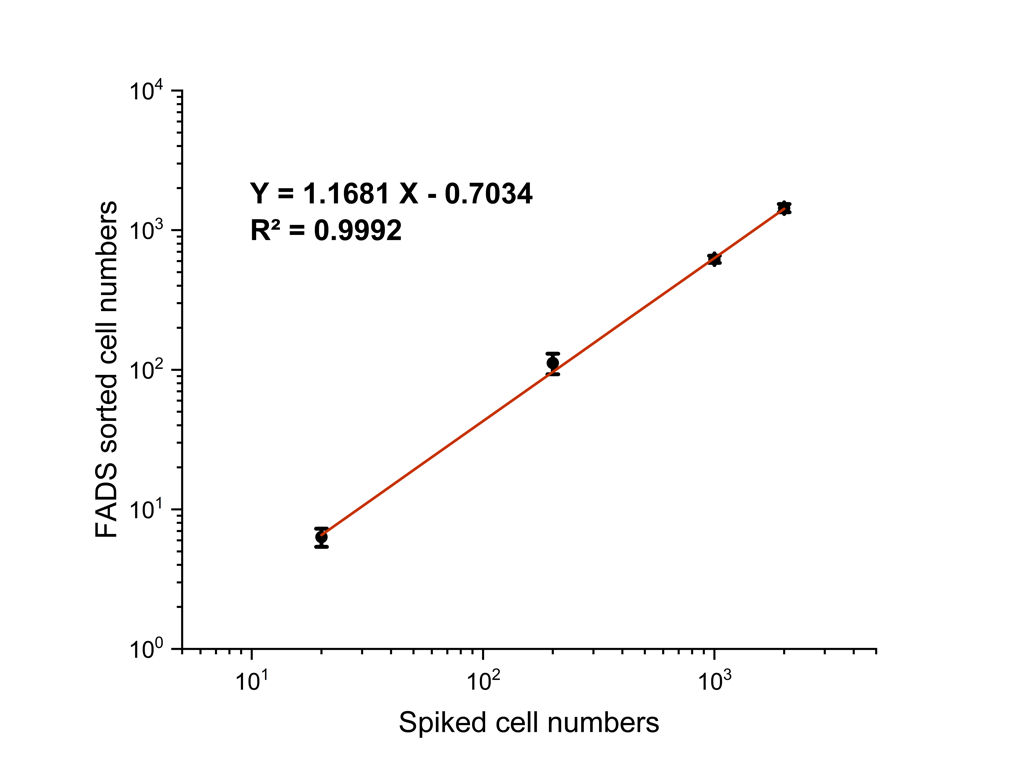


| **Name** | **Sequence（5’-3’）** |
| --- | --- |
| Aptamer | CACTACAGAGGTTGCGTCTGTCCCACGTTGTCATGGGGGGTTGGCCTG TTTTTTTTTTTTTTT GCTTATCAGACTGATGTTG |
| Aptamer-F | CACTACAGAGGTTGCGTCTGTCCCACGTTGTCATGGGGGGTTGGCCTG TTTTTTTTTTTTTTT GCTTATCAGACTGATGTTG-**FAM** |
| Tetra-A | ACATTCCTAAGTCTGAAACATTACAGCTTGCTACACGAGAAGAGCCGCCATAGTA TTTTTTTTTTTTTTT CAACATCAGTCTG ATAAGC |
| Tetra-B | TATCACCAGGCAGTTGACAGTGTAGCAAGCTGTAATAGATGCGAGGGTCCAATAC TTTTTTTTTTTTTTTT GACGAACTAGTT GATGAAGCTG |
| Tetra-C | TCAACTGCCTGGTGATAAAACGACACTACGTGGGAATCTACTATGGCGGCTCTTC TTTTTTTTTTTTTTTT GACGAACTAGTT GATGAAGCTG |
| Tetra-D | TTCAGACTTAGGAATGTGCTTCCCACGTAGTGTCGTTTGTATTGGACCCTCGCA TTTTTTTTTTTTTTTTT GACGAACTAGTTG ATGAAGCTG |
| SubstrateA-F | **FAM**-GTGTGCCTATTATGTCTCCTCCTGTGTGCCTATTATGTC TCCTCCT CAGCTTCATCAACTAGTT CGTCA |
| SubstrateA-Q | AACTAGTTGATGAAGCTG GACATAATAGGCACAC GACATA ATAGGCACAC-**BHQ1** |
| Assistant-A | GTGCCTATTATGTCGTGTGCCTATTATGTCCAGCTT |
| SubstrateB-F | AGGAGGA GACATAATAGGCACAC TGACGAACTAGTTG ATG AAGCTG-**FAM** |
| SubstrateB-Q | **TAMRA**-CAGCTTCATCAACTAGGTGTGCCTATTATGTCTC |
| Assistant-B | GCACACCTAGTTGATGAAGC |
| CK19-FP | GACTACAGCCACTACTACACGACCAT |
| CK19-RP | GAGCGGAATCCACCTCCACACT |
| EGFR-FP | CCAGTGACTGCTGCCACAACCA |
| EGFR-RP | CGCCGTCTTCCTCCATCTCATAGC |

**Table S1.** Sequences for oligonucleotide used in this work

**Table S2.** Comparation of current FADS-based mammalian cell sorting work to THE-FADS in this work^1-5^.


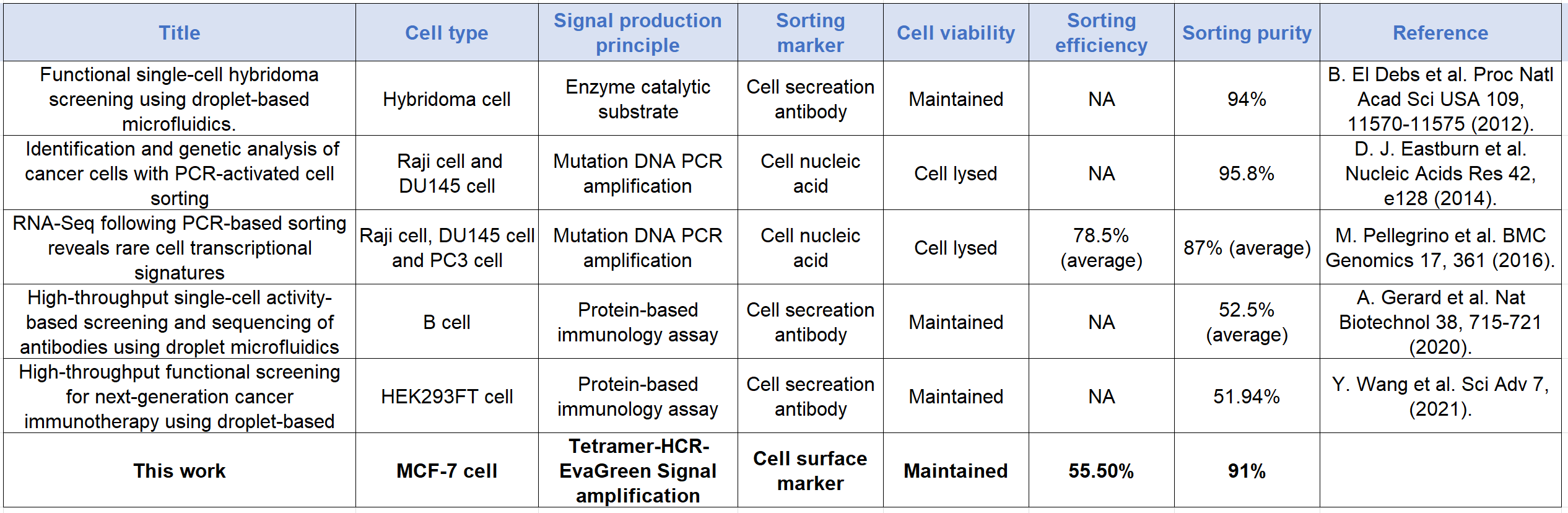


**References:**

1. Eastburn, D. J.; Sciambi, A.; Abate, A. R., Identification and genetic analysis of cancer cells with PCR-activated cell sorting. *Nucleic Acids Res* **2014,** *42* (16), e128.

2. El Debs, B.; Utharala, R.; Balyasnikova, I. V.; Griffiths, A. D.; Merten, C. A., Functional single-cell hybridoma screening using droplet-based microfluidics. *Proc Natl Acad Sci U S A* **2012,** *109* (29), 11570-5.

3. Gerard, A.; Woolfe, A.; Mottet, G.; Reichen, M.; Castrillon, C.; Menrath, V.; Ellouze, S.; Poitou, A.; Doineau, R.; Briseno-Roa, L.; Canales-Herrerias, P.; Mary, P.; Rose, G.; Ortega, C.; Delince, M.; Essono, S.; Jia, B.; Iannascoli, B.; Richard-Le Goff, O.; Kumar, R.; Stewart, S. N.; Pousse, Y.; Shen, B.; Grosselin, K.; Saudemont, B.; Sautel-Caille, A.; Godina, A.; McNamara, S.; Eyer, K.; Millot, G. A.; Baudry, J.; England, P.; Nizak, C.; Jensen, A.; Griffiths, A. D.; Bruhns, P.; Brenan, C., High-throughput single-cell activity-based screening and sequencing of antibodies using droplet microfluidics. *Nat Biotechnol* **2020,** *38* (6), 715-721.

4. Pellegrino, M.; Sciambi, A.; Yates, J. L.; Mast, J. D.; Silver, C.; Eastburn, D. J., RNA-Seq following PCR-based sorting reveals rare cell transcriptional signatures. *BMC Genomics* **2016,** *17*, 361.

5. Wang, Y.; Jin, R.; Shen, B.; Li, N.; Zhou, H.; Wang, W.; Zhao, Y.; Huang, M.; Fang, P.; Wang, S.; Mary, P.; Wang, R.; Ma, P.; Li, R.; Tian, Y.; Cao, Y.; Li, F.; Schweizer, L.; Zhang, H., High-throughput functional screening for next-generation cancer immunotherapy using droplet-based microfluidics. *Sci Adv* **2021,** *7* (24).
